# Dementia as Risk Factor for Severe Coronavirus Disease 2019: A Case-Control Study

**DOI:** 10.3389/fnagi.2021.698184

**Published:** 2021-06-29

**Authors:** Mariantonietta Pisaturo, Federica Calò, Antonio Russo, Clarissa Camaioni, Agnese Giaccone, Biagio Pinchera, Ivan Gentile, Filomena Simeone, Angelo Iodice, Paolo Maggi, Nicola Coppola

**Affiliations:** ^1^Infectious Diseases Unit, Department of Mental Health and Public Medicine, University of Campania Luigi Vanvitelli, Naples, Italy; ^2^Infectious Diseases Unit, Federico II University, Naples, Italy; ^3^Infectious Disease Unit, AORN Caserta, Caserta, Italy

**Keywords:** dementia, SARS-CoV-2, death, severity, COVID-19

## Abstract

**Background:**

The aim of the present study was to investigate the outcome of patients with SARS-CoV-2 infection and dementia.

**Patients and Methods:**

In a multicenter, observational, 1:2 matched case-control study all 23 patients with a history of dementia, hospitalized with a diagnosis of SARS-CoV-2 infection from February 28th 2020 to January 31st 2021 were enrolled. For each Case, 2 patients without dementia observed in the same period study, pair matched for gender, age (±5 years), PaO_2_/FiO_2_ (P/F) ratio at admission (<200, or >200), number of comorbidities (±1; excluding dementia) were chosen (Control group).

**Results:**

The majority of patients were males (60.9% of Cases and Controls) and very elderly [median age 82 years (IQR: 75.5–85) in the Cases and 80 (IQR: 75.5–83.75) in the Controls]. The prevalence of co-pathologies was very high: all the Cases and 43 (93.5%) Controls showed a Charlson comorbidity index of at least 2. During hospitalization the patients in the Case group less frequently had a moderate disease of COVID-19 (35 vs. 67.4%, *p* = 0.02), more frequently a severe disease (48 vs. 22%, *p* = 0.03) and more frequently died (48 vs. 22%, *p* = 0.03). Moreover, during coronavirus disease 2019 (COVID-19), 14 (60.8%) patients in the Case group and 1 (2.1%; *p* < 0.000) in the Control group showed signs and symptoms of delirium.

**Conclusion:**

Patients with dementia are vulnerable and have an increased risk of a severe disease and death when infected with COVID-19.

## Introduction

The novel coronavirus SARS-CoV-2, first identified in China on 31 December 2019, has rapidly spread around the world causing a global pandemic with over two million deaths.

The clinical presentation of the coronavirus disease 2019 (COVID-19) is variable, ranging from an asymptomatic infection to mild and more severe progressive respiratory failure (Macera et al., 2020; Cascella et al., 2021). Several risk factors for poor outcomes and mortality have been identified, such as age, hypertension, obesity, diabetes, and cancer (Gautret et al., 2020; Marfella et al., 2020; Onder et al., 2020; Zhou et al., 2020; Fedeli et al., 2021; Monari et al., 2021).

Older adults are particularly susceptible to COVID-19 infection due to the presence of multiple comorbidities and chronic diseases (Wynants et al., 2020). Moreover, the cognitive decline due to dementia, such as Alzheimer’s disease, exposes elderly subjects to a greater risk of becoming infected with COVID-19 (Korczyn, 2020); in fact, the poor adherence to infection control measures (e.g., hand washing, social distancing, and wearing masks) and their close physical contact with caregivers are risk factors for SARS-CoV-2 infection (Canevelli et al., 2020a). Furthermore, they often show an atypical clinical presentation (Bianchetti et al., 2020; Isaia et al., 2020; Ward et al., 2020) that may delay diagnosis and appropriate treatment and consequently impact their prognosis and survival (Alonso-Lana et al., 2020). Moreover, in the case of respiratory failure, the compliance with oxygen (O_2_) treatment with non-invasive or invasive ventilation is very low, with a possible poor prognosis.

Few data have been published on the impact of SARS-CoV-2 infection in patients with dementia (Canevelli et al., 2020b; Caratozzolo et al., 2020; Burns et al., 2021; Tsapanou et al., 2021; Wang et al., 2021; West et al., 2021). Although results are controversial, a worse outcome has been described among these patients (Hariyanto et al., 2020; Liu et al., 2020; McMichael et al., 2020). However, being older the patients with dementia had multiple comorbidities, so the nature of the association between dementia and poor prognosis of COVID-19 without the evaluation of age and co-pathologies associated has not yet been clearly evaluated.

The aim of the present pair-matched case-control study was to investigate the outcome of patients with SARS-CoV-2 infection and dementia, compared with patients without dementia but of the same age, presence of co-morbidities and clinical presentation at hospitalization, in order to assess its impact on the mortality and severity of the disease.

## Patients and Methods

### Study Design and Setting

We performed a multicenter, observational, 1:2 matched case-control study involving three COVID-19 Units in two cities in the Campania region in southern Italy, Naples and Caserta.

The patients enrolled were adults (≥18 years), hospitalized with a diagnosis of SARS-CoV-2 infection confirmed by a positive reverse transcriptase-polymerase chain reaction (RT-PCR) on a naso-oropharyngeal swab. Viral RNA was extracted by naso-oropharyngeal swab with QIAamp Viral RNA Kits (Qiagen GmbH, Hilden, Germany); the detection of SARS-CoV-2 was performed by RT-PCR test using Bosphore^®^ Novel Coronavirus (Anatolia Diagnostics and Biotechnology Products Inc., İstanbul, Turkey) Detection Kit V3, by primers designed on three viral regions: E, ORF1ab, and N regions.

The study period was from February 28th 2020 to January 31st 2021. All the patients with a diagnosis of dementia observed in the study period in one of the three centers participating were enrolled as Cases (Case group). For each Case, two patients without dementia observed by the same centers in the same study period, pair matched for gender, age (±5 years), PaO_2/_FiO_2_ (P/F) ratio at admission (<200, or >200), number of comorbidities (±1; excluding dementia) were chosen (Control group).

All demographic, clinical and laboratory data of both Cases and Controls were collected in a database. From this database we extrapolated the data.

The study was approved by the Ethics Committee of the University of Campania L. Vanvitelli, Naples (*n*°10877/2020). All procedures performed in this study were in accordance with the ethics standards of the institutional and/or national research committee and with the 1964 Helsinki declaration and its later amendments or comparable ethics standards. Informed consent was obtained from all participants included in the study.

### Variables and Definitions

The microbiological diagnosis of SARS-CoV-2 infection was defined as a positive RT-PCR test on a naso-oropharyngeal swab.

Dementia was diagnosed according to the clinical history of the patients.

The P/F ratio was considered as the arterial partial pressure of oxygen (aPP O_2_) investigated through hemogas analysis divided by the fraction of inspired oxygen concentration (FiO_2_) at the time of hospital admission.

The presence of underlying chronic diseases were defined according to the Charlson age-comorbidity index (Charlson et al., 1987), while medical conditions at risk of clinical deterioration were defined through a modified Early Warning Score (MEWS) (Subbe et al., 2001).

We defined patients with a mild, moderate or severe disease according to the clinical presentation of COVID-19. Precisely, patients with a mild infection did not need O_2_ therapy and/or had a MEWS score below 3 points. Patients with a moderate infection required low flow O_2_ therapy or non-invasive O_2_ therapy and/or had a MEWS score equal to or above 3 points (≥3). Lastly, patients with a severe infection needed management in an intensive care unit (ICU) and/or mechanical ventilation; in this definition we also included patients who died because of respiratory failure, multi-organ failure or septic shock. Patients were followed until SARS-CoV-2-RNA negativity at naso-oropharyngeal swab or discharge from hospital.

### Statistical Analysis

For the descriptive analysis, categorical variables were presented as absolute numbers and their relative frequencies. Continuous variables were summarized as mean and standard deviation if normally distributed or as median and interquartile range (IQR) if not normally distributed. We performed a comparison of patients with dementia and without dementia using Pearson chi-square or Fisher exact test for categorical variables and Student’s *t*- or Mann-Whitney tests for continuous variables.

A *p*-value below 0.05 was considered statistically significant. Analyses were performed by STATA.

## Results

During the study period, overall 672 patients with SARS-CoV-2 infection were observed in the three centers participating in the study. Of these 672 patients enrolled, 23 had a pre-existing diagnosis of dementia before the development of COVID-19 and were included in the Case group. Among the 649 patients observed without dementia, 46 were chosen as the Control group.

Of the 23 patients in the Case group, nine had a history of senile dementia, six of vascular dementia, five of Alzheimer’s disease, one of frontotemporal dementia, one of Parkinson dementia, and one of human immunodeficiency virus (HIV)-related dementia. Twelve patients in the Case group were in chronic treatment (Table 1): memantine in two, dopaminergic drug in two, benzodiazepine in one, selective serotonin reuptake inhibitors in one, antiepileptic drug in one, antipsychotic drug and benzodiazepine in one, antipsychotic drug and NMDA receptor antagonist in one, antipsychotic drug and gabapentin in one, antipsychotic drug and lithium in one; antipsychotic drug and acetylcholinesterase inhibitors in one. Eight patients had a history of delirium before COVID-19 that required pharmacological treatment.

**TABLE 1:** The therapies of the patients in case group.

Pts	Dementia drugs followed at home (previous hospitalization)	Need to increase therapy during hospitalization (0: not, 1: yes)	Need to add antipsychotic therapy during hospitalization (0: not, 1:yes)	Need to add benzodiazepine therapy during hospitalization (0: not, 1:yes)	Need to add opioids during hospitalization (0:not,1:yes)	Need to add other neuro/psychiatric drugs during hospitalization
1	//	0	0	0	0	No
2	Selective serotonin reuptake inhibitors	0	1	1	0	No
3	Antiepileptic drug	0	0	0	0	No
4	Benzodiazepine, antipsychotic drug	0	0	0	0	No
5	//	0	0	0	0	No
6	NMDA receptor antagonist, antipsychotic drug	0	0	0	0	No
7	Dopaminergic drug	0	0	0	0	No
8	Antipsychotic, gabapentin	0	0	0	0	No
9	Dopaminergic drug	1	1	0	0	No
10	Benzodiazepin	1	1	1	0	Antiepileptic drug (valproate)
11	//	0	0	1	0	No
12	//	0	1	0	0	Nr
13	//	1	0	0	0	No
14	Memantine	1	1	0	0	Nr
15	Lithium, antipsychotic drug	1	0	0	0	No
16	Antipsychotic drug, dopaminergic drug	0	0	1	0	No
17	//	1	0	1	0	No
18	//	1	0	1	0	No
19	//	1	0	1	0	No
20	//	0	0	1	0	No
21	//	0	0	1	0	Nr
22	Memantine	0	0	0	0	Nr
23	//	1	1	1	0	No

*Pts, patients number; //, no treatment required at home; Nr, not reported; NMDA, *N*-Methyl-D-aspartate.*

Table 2 shows the epidemiological and clinical characteristics of the Cases and Controls. There were no statistically significant differences in age, gender, and co-morbidities among COVID-19 patients with and without dementia. The majority of patients were males (60.9% of Cases and Controls) and very elderly [median age 82 years (IQR: 75.5–85) in the Cases and 80 (IQR: 75.5–83.75) in the Controls] (Table 2). The prevalence of co-pathologies was very high: all the Cases and 43 (93.5%) Controls showed a Charlson comorbidity index of at least 2; moreover, the median Charlson comorbidity index was similar in the two groups of patients [median 6 (IQR: 5–7) in Case group vs. 6 (IQR: 4–6) in the Control group] (Table 2). However, the patients in the Control group more frequently showed as underlying chronic diseases arterial hypertension (86.9 vs. 56.8%, *p* = 0.004).

**TABLE 2:** Demographic and clinical characteristics of the patients according to the presence or absence of dementia.

	With dementia	Without dementia	*p*-value
*N*° of patients	23	46	
Males, *N*° (%)	14 (60.9%)	28 (60.9%)	1
Age, years, median (IQR)	82 (75.5;85)	80 (75.5; 83.75)	0.62
*N*° (%) of patients in different age classes (years)			
40–49	0	1 (2,3%)	1
50–59	2 (8.7%)	3 (6.5%)	1
60–69	1 (4.3%)	3 (6.5%)	1
70–80	5 (21.7%)	17 (36.9%)	0.27
>80	15 (65.2%)	22 (47.8%)	0.17
Charlson comorbidity index, median (IQR)	6 (5;7)	6 (4;6)	0.06
*N*° (%) of patients with Charlson index ≥ 2	23 (100%)	43 (93.5%)	0.54
*N*° (%) of patients with an underlying chronic disease (dementia)	23	44	0.55
With hypertension	13 (56.8%)	40 (86.9%)	**0.004**
With cardio-vascular disease	11 (47.8%)	21/45.6%)	0.86
With diabetes	4 (17.4%)	17 (36.9%)	0.16
With chronic obstructive pulmonary disease	8 (34.8%)	15 (32.6%)	0.86
With liver cirrhosis	0	2 (4.3%)	0.55
With malignancy	2 (8.7%)	6 (13%)	0.71

*Bold value indicate our way to highlight statistically significant p-value.*

Table 3 shows the data on the clinical presentation of COVID-19 in the Cases and Controls. No statistically significant differences were found at admission between P0_2_ [median 69.5 mmHg (IQR: 56.3–79) in the Cases and 65 (IQR: 59.5–74. 5) in the Controls] and P/F [median 244.5 (IQR: 169.7–320.5) in the Cases and 245 (IQR: 205–290) in the Controls] (Table 3).

**TABLE 3:** Clinical presentation of coronavirus disease 2019 (COVID-19) in case and control groups.

	With dementia	Without dementia	*p*-value
*N*° of patients	23	46	
PA02 (mmHg) at admission, median (IQR)	69.5 (79,563)	65 (74.5–59.5)	0.46
P/F at admission, median (IQR)	244.5 (320.5–169.75)	245 (290–205)	0.81
*N*° (%) of patients with the worst respiratory support:			
No respiratory support or need for nasal cannula	9 (39.13)	23(50)	0.39
With need for HFNC	9 (39.13)	9 (19.56)	0.08
With need for CPAP	1 (4.34)	8 (17.39)	0.25
With need for NIV	4 (17.4)	6 (13)	0.72
With need for invasive ventilation	10 (43.5)	10 (21.7)	0.06
*N*° (%) of patients with mild disease	4 (17.4)	7 (15.2)	1
*N*° (%) of patients with moderate disease	8 (34.8)	29 (63)	**0.02**
*N*° (%) of patients with severe disease	11 (47.8)	10 (21.7)	**0.03**
*N*° of deaths (%)	11 (47.8)	10 (21.7)	**0.03**
Days form admission to discharge, median (IQR)	20(12–45)	20(15–31)	0.63
Days from admission to death, median (IQR)	12(9–21)	19(12.5–30)	0.34

*IQR, interquartile range; HFNC, high flow nasal cannulas; CPAP, continuous positive airway pressure; NIV, non-invasive ventilation. Bold value indicate our way to highlight statistically significant p-value.*

As regards the most serious respiratory support needed during hospitalization, a similar prevalence was found in high flow nasal cannulas (HFNC) [9 (39.1%) vs. 9 (19.6%; *p* = 0.08] in continuous positive airway pressure (CPAP) [1 (4.3%) vs. 8 (17.4%); *p* = 0.25] in non-invasive ventilation (NIV) [4 (17.4%) vs. 6 (13%); *p* = 0.72]. In the Case group, the prevalence of patients needing invasive ventilation was higher [10 (43.5) vs. 10 (21.7); *p* = 0.06], but with a difference not significant to the statistical analysis (Table 2). However, during hospitalization, with respect to the patients in the Control group, those in the Case group less frequently had a moderate disease of COVID-19 (35 vs. 67.4%, *p* = 0.02), more frequently a severe disease (48 vs. 22%, *p* = 0.03) and more frequently died (48 vs. 22%, *p* = 0.03) (Table 2). Moreover, although no difference between the two group of patients was observed in time from admission to discharge, the patients with dementia had a shorter period between admission and death [median and IQR of 12 (9–21) days vs. 19 (12.5–30) days], a difference without statistical significance (Table 3).

During COVID-19, 14 (60.8%) patients in the Case group and 1 (2.1%; *p* < 0.000) in the Control group showed signs and symptoms of delirium and required the addition of drugs to control these (Table 1): antipsychotic drug in three, benzodiazepine in seven and both in three patients in the Case group; the only patient in the Control group showing signs of delirium required the addition of an antipsychotic drug and benzodiazepine.

In the Case group, no difference in mortality was observed between the 14 patients with signs and symptoms of delirium during COVID-19 and the nine without (35.7 vs. 66.6%, *p* = 0.21).

## Discussion

In the present 1:2 case-control study performed in three COVID-19 Units in southern Italy we found that the patients with pre-existing dementia showed a worse prognosis of COVID-19. They more frequently showed a severe clinical outcome and more frequently died than those without dementia, but showed a similar age, number of pre-existing co-pathologies and respiratory failure at admission.

We know that globally more than 50 million people have dementia that has emerged as a pandemic in an aging society (Fox and Petersen, 2013; Alzheimer’s Disease International, 2019). Thus, the double hit of dementia and COVID-19 pandemics has raised great concern.

A meta-analysis on 24 studies with 46,391 dementia patients showed that dementia was associated with severe COVID-19 [RR 2.63 (95% CI 1.41–4.90), *p* = 0.002] and mortality from COVID-19 infection [RR 2.62 (95% CI 2.04–3.36), *p* < 0.00001] (Hariyanto et al., 2020). However, the data available in the literature on this topic cannot be considered conclusive. In fact, since the patients with dementia were very elderly, they had a lot of co-pathologies. Thus, the impact of age and the presence of co-pathologies in the clinical presentation of patients with dementia have not been clearly analyzed. For example, Bianchetti et al. (2020) showed that the mortality rate was higher (62.2%) among 82 patients suffering from dementia than that (26.2%) observed in 545 without. Instead, the 82 patients with dementia were older (mean age of 82.6 ± standard deviation 5.3) than the 545 without (68.9 ± 12.7), with no analysis of the presence of co-pathologies.

Interestingly, the majority (69.5%) of patients with dementia in the present study during COVID-19 showed symptoms that required the addition of antipsychotic or benzodiazepine drugs. Thus, as already suggested by other authors (Kales et al., 2019), the SARS-CoV-2 patients with dementia who need hospital care represent a challenge for COVID-19 units and an increase in stress to manage non-compliant patients and with behavioral problems. In fact, delirium caused by hypoxia, a prominent clinical feature of COVID-19, can complicate the presentation of dementia (Marcantonio, 2017) and increase the suffering of people with dementia hospitalized for COVID-19, as well as the cost of medical care and the need for dementia support.

Although without a difference in the statistical significance probably due to the small number of patients enrolled, it seems interesting that the patients with dementia had a shorter period between admission and death compared with those without. These data are in agreement with the observation of the Italian Institute of Health: considering the data on the 2,621 deaths due to COVID-19 in Italy, the patients with dementia showed a more rapid clinical worsening compared with individuals with intact cognition (Canevelli et al., 2020a).

The factors involved in the association between dementia and worse prognosis of COVID-19 could be many. Of course, the patient’s lack of cooperation in performing the main therapy for SARS-CoV-2 pneumonia could be one of the reasons for the negative outcome of the disease in these patients. Then, the neurotropism of the virus and the presence of angiotensin-converting enzyme 2 (ACE-2) receptor, the cellular receptor for the SARS-CoV-2, on the brain and glial tissue makes the central nervous system a potential target for the virus (Yan et al., 2020; Barillari et al., 2021). The virus could infect the brain also through a disrupted blood-brain barrier that was often compromised in the aging brain and in neurodegenerative diseases, such as Alzheimer’s disease (Hascup and Hascup, 2020). In view of this, it is likely that neurological manifestations caused by the virus worsen the already damaged neurological function of patients with dementia, making the prognosis worse. Furthermore, some studies had shown how the ACE receptor polymorphisms could influence the prognosis of patients with COVID 19 and are associated with Alzheimer’s disease (Cao et al., 2020; Delanghe et al., 2020; Gómez et al., 2020).

Finally, severe COVID-19 outcomes are often associated with a “cytokine storm” (Castelli et al., 2020); so elderly individuals affected by dementia could be at a higher risk due to a higher baseline of inflammation that steadily increases with age (Rea et al., 2018; Naughton et al., 2020).

Our study shows several limits; first, the retrospective nature of the study; second, we evaluated only in-hospital mortality; third, the number of patients enrolled with dementia was low. The strengths of the study are the multicenter and case-control nature of the design, which makes it possible to look at multiple risk factors at the same time, especially age and the presence of co-pathologies.

In conclusion, patients with dementia are vulnerable and have an increased risk of serious morbidity, admission to ICUs, and death when infected with COVID-19. Thus, it is necessary to carry out an early diagnosis of SARS-CoV-2 infection in this population and to implement all measures to ensure proper management of the disease at home, with the use of telemedicine and digital technological devices, such as smart phones, which can be very useful in remote monitoring and care. Ideally, the use of monoclonal antibodies can be considered in these patients in an early phase to reduce the need of hospitalization and progression of the disease. In addition, it is necessary to establish a multidisciplinary team with an infectious disease specialist, a psychiatrist, a psychologist, social workers, nurses and volunteers to manage this difficult-to-treat-population. Finally, implementing the anti-COVID-19 vaccination in these patients is a priority.

## Data Availability Statement

The original contributions presented in the study are included in the article/supplementary material, further inquiries can be directed to the corresponding author.

## Ethics Statement

The studies involving human participants were reviewed and approved by AOU Vanvitelli. The patients/participants provided their written informed consent to participate in this study.

## Author Contributions

MP, FC, and NC were involved in study concept and design, and drafting of the manuscript. PM and IG were involved in critical revision of the manuscript for important intellectual content. CC, AR, AG, and BP were involved in acquisition of data, analysis and interpretation of data, and in critical revision of the manuscript. All authors contributed to the article and approved the submitted version.

## Conflict of Interest

The authors declare that the research was conducted in the absence of any commercial or financial relationships that could be construed as a potential conflict of interest.
